# Tauroursodeoxycholic acid regulates macrophage/monocyte distribution and improves spinal microenvironment to promote nerve regeneration through inhibiting NF-κB signaling pathway in spinal cord injury

**DOI:** 10.3389/fphar.2025.1554945

**Published:** 2025-04-10

**Authors:** Yonghui Hou, Yage Zhang, Lin Ma, Dan Luo, Wanshun Wang, Shunmei E., Chengxi Huang, Yu Hou, Shudong Chen, Jiheng Zhan, Liangliang Xu, Dingkun Lin

**Affiliations:** ^1^ Department of Orthopedic Surgery, Guangdong Provincial Hospital of Chinese Medicine, The Second Affiliated Hospital of Guangzhou University of Chinese Medicine, Guangzhou, Guangdong, China; ^2^ Lingnan Medical Research Center, Guangzhou University of Chinese Medicine, Guangzhou, Guangdong, China; ^3^ Key Laboratory of Orthopaedics & Traumatology, The First Affiliated Hospital of Guangzhou University of Chinese Medicine, Guangzhou University of Chinese Medicine, Guangzhou, China; ^4^ College of Physical Education and Health, Guangzhou University of Chinese Medicine, Guangzhou, China; ^5^ Department of Laboratory Medicine, Guangdong Provincial Hospital of Chinese Medicine, The Second Affiliated Hospital of Guangzhou University of Chinese Medicine, Guangzhou Higher Education Mega Center, Guangzhou, Guangdong, China; ^6^ Culver Academies, Culver, IN, United States

**Keywords:** tauroursodeoxycholic acid, spinal cord injury, monocyte, macrophage, microenvironment, nerve regeneration, inflammation

## Abstract

**Introduction:**

Following spinal cord injury (SCI), blood-borne monocytes infiltrate the spinal cord, differentiate into macrophages, and dominate the lesion site. Inflammatory responses mediated by macrophages determine nerve regeneration and functional recovery after SCI. Tauroursodeoxycholic acid (TUDCA) shows a neuroprotective effect in different SCI animal models. However, the underlying mechanism of TUDCA regulating monocytes/macrophages to impact nerve regeneration after SCI has not been elucidated clearly. This study aims to investigate the effect of TUDCA on monocyte/macrophage distribution and nerve regeneration in the subacute stage of SCI.

**Methods:**

Transwell analysis, Bromodeoxyuridine (BrdU) staining, and TUNEL staining were performed to evaluate the effect of TUDCA on regulating the inflammatory response to impact spinal neural stem cells (NSCs) proliferation and migration, spinal neuron survival, and axon degeneration in vitro. H&E staining, RNA sequencing, and a series of immunofluorescent staining were performed to investigate the pathological progress, gene expression changes, monocytes/macrophages distribution, and nerve regeneration after TUDCA treatment in SCI mice.

**Results:**

We found TUDCA restored spinal NSCs migration and proliferation and reduced spinal NSCs and neurons apoptosis and axon degeneration by regulating inflammatory response in vitro. TUDCA treatment promoted wound healing, down-regulated genes related to inflammatory response, and up-regulated genes related to spinal cord development in SCI mice.

**Conclusions:**

Our study provided evidence that TUDCA treatment regulated monocyte/macrophage distribution and improved the microenvironment to promote nerve regeneration in SCI mice.

## Introduction

Traumatic spinal cord injury (SCI) is a devastating disease caused by mechanical injury, which not only results in a second injury but also in prolonged progressive neurodegeneration (Cowan et al., 2020). SCI pathological progress is a multiple process that occurs temporarily (Milich et al., 2021). Following the blood-spinal cord barrier (BSCB) damage, peripheral myeloid cells mainly neutrophils and monocytes first infiltrate into the spinal cord, and the number of neutrophils in the spinal cord peaks around 1-day post-injury. Neutrophil’s presence in the spinal cord is short, however, neutrophil infiltration activates other immune cells and helps to initiate the inflammatory cascade by the release of chemokine and cytokines (Milich et al., 2019). At around day 3 post-injury, extravasated monocytes migrate to the injury site of the cord and begin to differentiate into macrophage that phagocytizes cellular debris and secret pro-inflammatory factors-induced neural and glial cell death (Zhu et al., 2015). Concurrently, astrocytes, oligodendrocyte progenitor cells, and neural stem cells (NSCs) are activated and begin to proliferate at the rim of the lesion site of the cord and to migrate into the epicenter of the damage (Li et al., 2024). On day 7 post-injury, the number of macrophages/monocytes reached their peak and dominated the lesion site of the cord. Meanwhile, the fibrotic scar gradually forms surrounded by an astroglial scar, which can help limit macrophage/monocyte infiltration and the expansion of the lesion area (Sofroniew, 2015). The number of NSCs also reaches its peak, and they give rise to astrocytes, oligodendrocytes, and neurons. Their fate is attributed to the microenvironment of the spinal cord (Stenudd et al., 2015a). By day 14 post-injury, the lesion site including the fibrotic scar and glial scar has reached a relatively stable state (Li et al., 2021). Therefore, the first 7 days after injury are highly dynamic and provide the optimal period of therapeutic intervention.

Tauroursodeoxycholic acid (TUDCA) is used for the treatment of cholestatic liver diseases in clinical. It can cross the blood-brain barrier and is not toxic (Zangerolamo et al., 2021). Recently, it has been reported to show neuroprotective roles in several models of neurodegeneration-related disease, including Parkinson’s disease (PD) (Cuevas et al., 2022), Alzheimer’s disease (AD) (Ochiai et al., 2021), and Huntington’s disease (HD) (Keene et al., 2002), based on its ability on the modulation of apoptosis, inflammation, oxidative stress, endoplasmic reticulum stress, and cell proliferation in animal models of the disease. At present, there are some studies about the neuroprotective action of TUDCA in SCI animal models (Smaling et al., 2023). It was reported that TUDCA shows cytoprotective effects in cellular cultures including neurons and glia, anti-inflammatory effects by reducing NF-κb pathway activation in macrophage and microglia cultures (Yanguas-Casás et al., 2017), and reduces apoptosis by activating Akt and cAMP signaling pathway (Dong et al., 2020). In the SCI animal model, TUDCA is demonstrated to reduce inflammatory cytokines such as iNOS and CD86 (Kim et al., 2018), suppress neuronal apoptosis, and improve functional recovery (Dong et al., 2015). Our previous study also demonstrated that TUDCA can reduce apoptosis, oxidative stress, and inflammatory response in the subacute stage, and promote axon regeneration and remyelination to improve functional recovery (Hou et al., 2021). Although most of the studies demonstrate that TUDCA shows a cytoprotective effect by reducing apoptosis and inflammatory response to improve functional recovery in the SCI animal model, the underlying mechanism of TUDCA on inflammatory response has not been elaborated clearly.

Excessive inflammatory response after SCI causes neuronal and glial cell death that leads to damage expansion, secrets cytokines, and chemokine and forms a microenvironment that is not conducive to axonal regeneration (Bloom et al., 2020). Therefore, reducing inflammatory response is an effective intervention in SCI treatment. Peripheral myeloid cells including neutrophils and monocytes infiltrate the spinal cord immediately after injury, and macrophage/monocyte dominates the lesion site around 7 days post-injury (Fu et al., 2023). The first 7 days after SCI are highly dynamic and there is an overwhelming release of pro-inflammatory cytokines at this stage which directly influences the SCI pathological progress (Mortezaee et al., 2018). And blood-borne macrophage/monocyte infiltration after injury is pivotal in this process. Therefore, this study aims to investigate the effect of TUDCA on macrophage/monocyte infiltration and distribution, scar formation, NSCs proliferation and migration, and nerve degeneration during the first 7 days to explore the underlying mechanism of TUDCA in SCI.

## Materials and methods

### Cell culture and treatment

RAW264.7 cells were cultured in Dulbecco’s modified Eagle medium (DMEM; Gibco) containing 10% fetal bovine serum (FBS; Gibco) and 1% penicillin/streptomycin at 37°C in a 5% CO_2_ incubator. Cells were passaged every 2 days. For the following experiments, cells were pretreat with TUDCA (200 μM and 400 μM) for 30 min, and treated with Lipopolysaccharide (LPS, 1 μg/mL) for 24 h.

### ELISA analysis

Cells were seeded into 6-well plates, and pretreated with TUDCA (200 μM and 400 μM) for 30 min, and then incubated with or without Lipopolysaccharide (LPS, 1 μg/mL) for 24 h. Supernatant for each treatment was collected, and the concentrations of IL-1β, IL-6 and TNF-α were quantified by ELISA according to manufacturer’s protocol.

### NSCs and neurons from spinal cord primary culture

Spinal cord NSCs and neurons were isolated from C57BL/6 mice E12.5 embryos cultured as described in our previous study (Hou et al., 2022). The spinal cords were dissected out using forceps under microscope, cut into small pieces using scissors, and then digested with 2 mg/mL trypsin (T4799; Sigma), and 100 μg/mL DNase I (11284932001; Roche) in DMEM at 37°C for 25 min. After that, DMEM with 10% FBS was used to terminate the digestion. The single cell suspension was filtered by a 40 μm cell strainer (BD Falcon) and centrifuged at 800 rpm for 5 min. For NSCs culture, cell pellet was suspended in Dulbecco’s modified Eagle medium: F-12 (DMEM/F12 medium; Gibco) containing 2% B27 (B-27™ Supplement; Gibco), 10 ng/mL FGF2 (Peprotech), 10 ng/mL EGF (Peprotech), and seeded into 60 mm uncoated petri dishes. Half of the culture medium was refreshed every 2 days afterwards. For the following experiments, the neuropheres were digested into single cells, and seeded into poly-D-lysine pre-coated plates.

For spinal cord neuron culture, cell pellet was suspended in DMEM with 10% FBS, and seeded into 6-well plates or 24-well plates pre-coated with poly-D-lysine. 4–6 h later, cells were cultured in neuron culture medium containing Neurobasal medium (Gibco), 2% B27, 0.5 mM L-Glutamine (Gibco). Half of the medium was refreshed every 2 days.

To investigate the effect of TUDCA on spinal NSCs and neurons survival under inflammatory response *in vitro*, 50 μL supernatant for each treatment of RAW264.7 cell culture was added into spinal cord NSCs or neurons culture in 24-well plates, and co-cultured for another 24 h.

### Transwell migration

The spinal NSCs migration assay was performed using a Transwell chamber (Corning^®^ Transwell^®^ 24 well plates). In the migration assay, 2 × 10^4^ spinal NSCs suspended in DMEM/F12 medium were seeded into the upper chamber, and 600 μL RAW264.7 cell culture supernatant induced with LPS and treated with or without TUDCA were added to the bottom of the chamber. 24 h later, the cells were fixed with 4% paraformaldehyde (PFA) for 15 min and stained with 0.1% crystal violet for 15 min. The migrated cells were captured under an Olympus IX73 microscope and quantified using ImageJ software.

### Bromodeoxyuridine labeling

At the end of culture, 10 μM Bromodeoxyuridine (BrdU) (B5002; Sigma) was added into the medium and incubated at 37°C for another 1 h. Then cells were fixed in 4% PFA for 30 min and incubated with 2N HCl at 37°C for 5 min to expose BrdU within proliferating cells. And the rest of BrdU labeling was performed as the procedure for the immunofluorescence staining. All the images were taken under a fluorescence microscope (Olympus IX73).

### TUNEL assay

Apoptotic cells were identified by TUNEL staining using One Step TUNEL Apoptosis Assay Kit (Beyotime) according to manufacturer’s protocol. The number of the apoptotic cells and the total number of cells were counted.

### Sholl analysis

Sholl analysis was performed to analyze the number of dendritic branch intersections at the given distance from the soma using ImageJ software as previously described (Bird and Cuntz, 2019). Statistics were calculated using one-way ANOVA with Bonferroni’s multiple comparisons test.

### Animals, SCI model, and treatment

C57BL/6 male mice, weighing 20–25 g for 10 weeks, were provided by Guangdong Medical Experimental Animal Centre. Estrogens and estrogenic compounds has been reported to effectively mitigate the effects of SCI to improve functional recovery (Shvetcov et al., 2023). Male mice were used to construct the SCI animal model in this study. All animal experiments were approved by the Ethics Committee of Guangzhou University of Chinese Medicine and performed under the guidelines of the Chinese National Institutes of Health (Guangzhou, China, Certificate No. 20230407006).

The contusive SCI model to mice was performed as previously described (Hou et al., 2021) under sterile conditions. Briefly, mice were anesthetized with 3% pentobarbital sodium (50 mg/kg) by intraperitoneal injection. Laminectomy was performed to fully expose the T10 spinal cord. A pneumatic impact device (Model PCI3000, RWD Life Science Co., Ltd.) controlled by a computer was used to deliver the contusion injury as our previous study described. It was observed that the hit portion of the spinal cord became congested and turned red, and the mice exhibited transient spasmodic twitches in their tails and hind limbs, indicating that the SCI model was successfully established. After surgery, bladders were expressed manually twice a day until the injured mice could urinate normally. A total of 60 mice were randomly divided into three groups, sham group, SCI group, and TUDCA group. Laminectomy was only performed on mice in the sham group without contusion injury. Referring to our previous study, TUDCA at 200 mg/kg dosage was given to the mice in the TUDCA group by oral administration once a day until the end of the experiment. Meanwhile, the mice in sham and SCI groups were given an equal volume of normal saline solution once a day.

### Functional behavior evaluation

Basso-Beattie-Bresnahan (BBB) rating scale at different time points (day 3, day 7, and day 14) and footprint test at day 14 were used to evaluate hind limb motor function. Based on the movement and coordination of the hind limb joints, the BBB score ranges from 0 to 21 (0 = complete paralysis, 21 = normal gait). The footprint test was carried out by soaking the hind limbs of mice with red dye. The footprints are scanned and the digital images are used to analyze their gait.

### Tissue preparation

Mice were anesthetized with 3% pentobarbital sodium as described above. For the following immunofluorescence staining and pathological analysis, mice were transcardially perfused with PBS and fixed with 4% paraformaldehyde (PFA) in 0.1 M PBS for another 2 days at room temperature. Then the spinal cord tissues were dissected out, gradually dehydrated by 70%, 80%, 95%, and 100% ethanol, and embedded in paraffin with appropriate orientation. The embedded spinal cords were sectioned at 5-μm thickness by microtome centered on the injured area.

### RNA sequencing

For RNA sequencing analysis, the spinal cord tissues around the lesion epicenter (±0.2 cm) were isolated under anesthetization on day 7 after SCI, and then homogenized in NucleoZOL (MNG, Shanghai, China). Total RNA was extracted following the manufacturer’s protocol and qualified using Fragment Analyzer before RNA sequencing. RNA sequencing was performed by BGI Genomics Company (Shenzhen, China). Heatmap cluster and volcano plots were generated by the pheatmap (version 1.0.12) and ggplot2 (version 3.5.1) packages of Heatmap and Volcano. DAVID database and Venny 2.1 software were used to analyze functional annotation enrichment and identify the core gene associated with SCI and regulated by TUDCA treatment.

### Immunofluorescence staining on paraffin sections and cells

The sections were dewaxed in xylene two times each for 10 min and rehydrated with 100%, 95%, 80%, and 70% ethanol solution each for 5min and in tap water for 5min. Then heat-mediated antigen retrieval was performed on sections using sodium citrate buffer (10 mM citrate pH 6.0). After blocking no-specific staining with blocking buffer (10% normal horse serum and 0.2% Triton X-100 in PBS) at room temperature for 1h, sections or cells were incubated with different primary antibodies diluted in blocking buffer at 4°C overnight. The list of primary antibodies was as follows rabbit anti-CD68 (1:300; Boster Biological Engineering Co.), rabbit anti-iNOS (1:200, Boster Biological Engineering Co.), rabbit anti-CD11b (1:100; ab133357; Abcam), mouse anti-GFAP (1:300; Boster Biological Engineering Co.), mouse anti-Nestin (1:200; ab6142, Abcam), rabbit anti-glial fibrillary acidic protein (GFAP; 1:300; Boster Biological Engineering Co.), rabbit anti-NeuN (1:200; ABclonal Biotechnology Co.), mouse anti-beta III tubulin (Tuj1; 1:200; MAB1637, Millipore), mouse anti-microtubule-associated protein (MAP2; 1:200; Boster Biological Engineering Co.), mouse anti-BrdU (1:200, Cell Signaling Technology). Alexa Fluor™ 488 or 555-conjugated anti-rabbit or anti-mouse secondary antibodies were used to recognize primary antibodies. Finally, sections were visualized with Pannoramic MIDI (3D HISTECH Ltd.)

### Statistical analysis

Statistical analyses were performed using SPSS 16.0 software (SPSS Inc.). All data were presented as means ± SEM. Student’s t*-*test (normal distribution) or Wilcoxon Mann–Whitney test (non-normal distribution) was performed to compare the two groups. One-way ANOVA (normal distribution) or Kruskal–Wallis test (non-normal distribution was performed for multiple comparisons. A *p* value less than 0.05 was considered to indicate statistically significant (expressed as *p < 0.05 or **p < 0.01).

## Results

### TUDCA reduced inflammation to restore spinal NSCs migration and proliferation and prevent cell apoptosis *in vitro*

**FIGURE 1:**
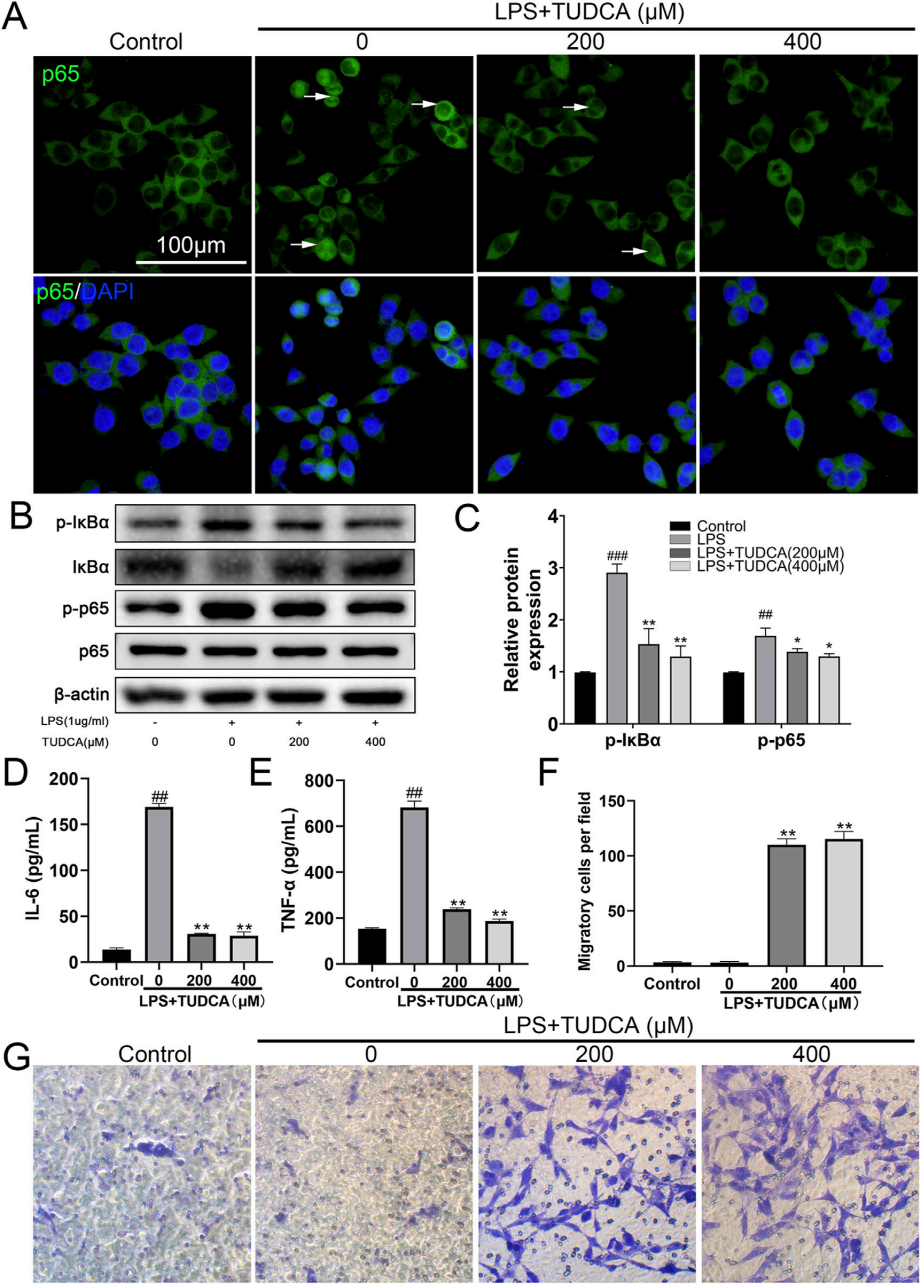
TUDCA reduced inflammatory response and restored spinal NSCs migration *in vitro*. **(A)** Immunofluorescence staining showing the nuclear translocation of p65 (green) in LPS-induced RAW267.4 cells after TUDCA treatment. **(B, C)** Western blot analysis and relative quantification showing the expression levels of NF-κB signaling pathway related proteins (p-IκBα, IκBα, p-p65 and p65) in LPS-induced RAW264.7 cells after TUDCA treatment. **(D, E)** IL-6 and TNF-α expression were determined using ELISA analysis in LPS-induced RAW264.7 cell culture supernatant after TUDCA treatment. **(F, G)** Comparison of spinal NSCs migration co-cultured with LPS-induced RAW264.7 cell culture supernatant with or without TUDCA treatment. All experiments were performed in triplicated and data were presented means ± SEM, *n* = 3 per group. ^*###*^ *P < 0.001* vs. Control*,* ^*##*^ *P < 0.01* vs. Control, **P < 0.01 vs. LPS-induced group, *P < 0.05 vs. LPS-induced group.

**FIGURE 2:**
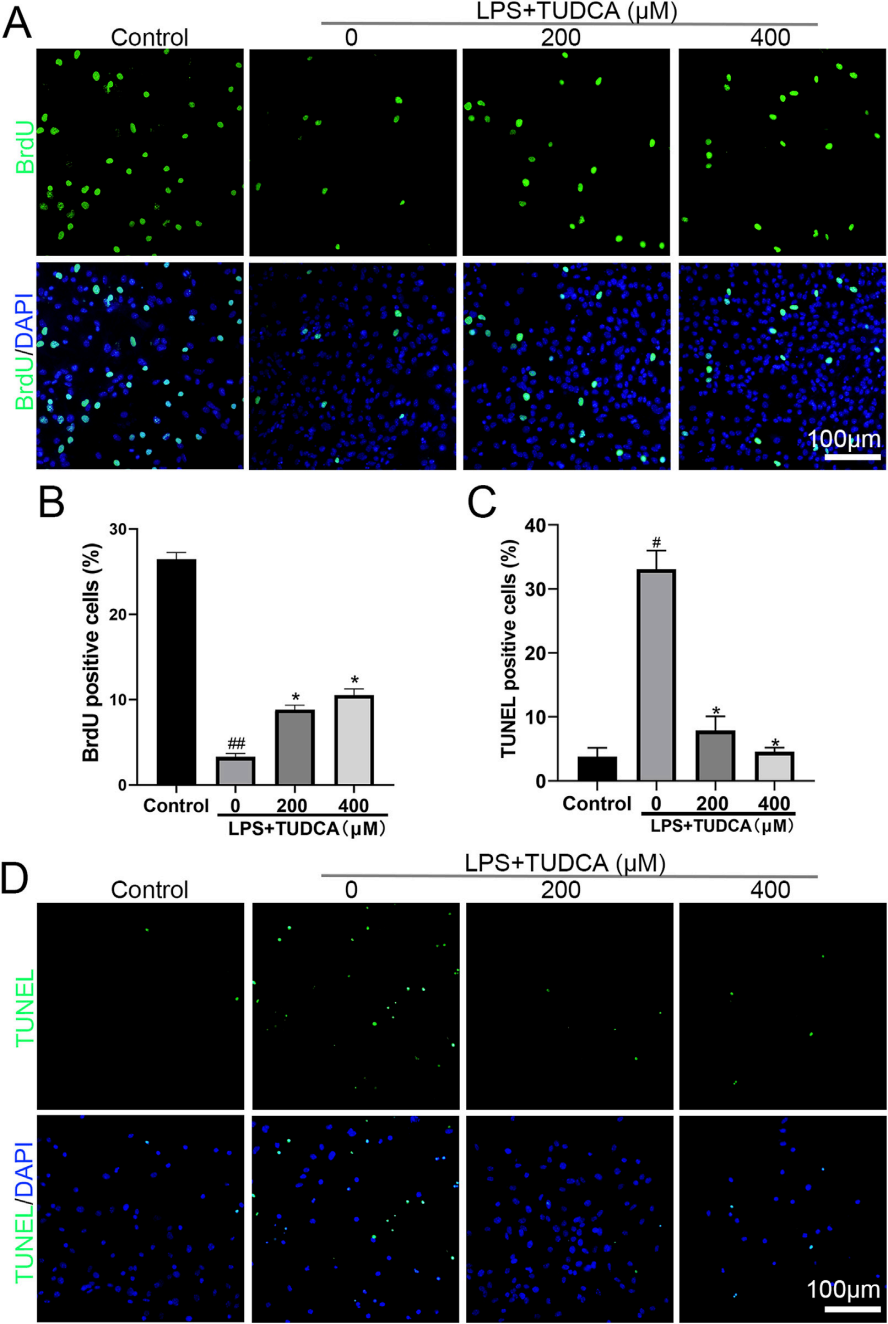
TUDCA restored spinal NSC proliferation and showed cytoprotection by regulating inflammatory response. **(A, B)** BrdU labeling analysis on spinal NSCs proliferation. **(C, D)** TUNEL staining analysis on spinal NSCs apoptosis. All experiments were performed in triplicated and data were presented means ± SEM, *n* = 3 per group. ^*#*^ *P < 0.05* vs. Control, ^*##*^ *P < 0.01* vs. Control,*P < 0.05 vs. LPS-induced group.

Following SCI, ependymal cells considered as endogenous NSCs become activated, proliferate, and migrate towards the lesion site, providing a potential possibility for neuron regeneration. The inflammation microenvironment is crucial for the fate of endogenous NSCs after SCI (Covacu and Brundin, 2017). Therefore, we investigated the effect of TUDCA on spinal NSC migration, proliferation, and apoptosis with an inflammatory culture *in vitro*. TUDCA has been reported to inhibit neuroinflammation by inhibiting NF-κB signaling pathway (Wu et al., 2019). We also proved that TUDCA reduced nuclear translocation of p65 (Figure 1A), downregulated p-IκBα and p-p65 (Figures 1B, C) and inhibited IL-6 and TNF-α (Figures 1D, E) expression in LPS-induced RAW264.7 cells. Transwell results showed that few spinal NSCs were observed when cultured with RAW264.7 cell culture supernatant induced with or without LPS, whereas the cell number of migratory cells increased after TUDCA treatment (Figures 1F, G). BrdU labeling analysis showed that many more spinal NSCs were BrdU positive after TUDCA treatment as compared with the LPS-induced group (Figures 2A, B). Moreover, the number of TUNEL-positive cells increased after induced with LPS compared with the control group, but after TUDCA treatment a few cells were TUNEL positive (Figures 2C, D). In summary, TUDCA treatment could restore spinal NSCs migration and proliferation, and prevent their apoptosis by reducing inflammatory response.

### TUDCA prevented spinal neuron apoptosis and alleviated axon degeneration *in vitro*

**FIGURE 3:**
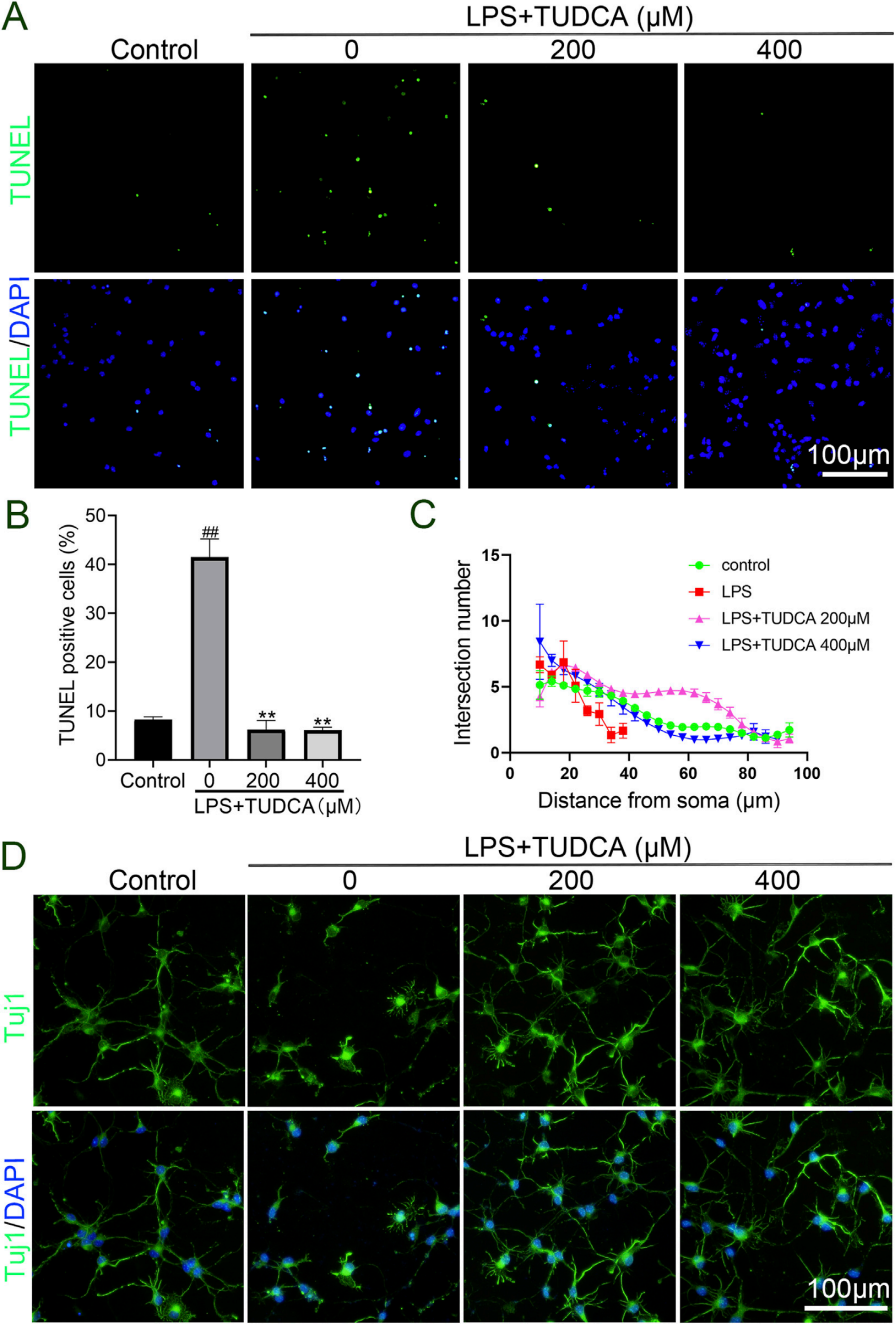
TUDCA alleviated spinal neuron apoptosis and axon degeneration byregulating inflammatory response. **(A, B)** TUNEL staining analysis on spinal neuron apoptosis. **(C, D)** Sholl analysis of the axon from the cell body and immunofluorescence images showing the axon labeled with Tuj1 in spinal neurons. All experiments were performed in triplicated and data were presented means ± SEM, *n* = 3 per group. ^*##*^ *P < 0.01* vs. Control, **P < 0.01 vs. LPS-induced group.

Excessive inflammatory response has been reported to cause neuronal and glial cell death and axon degeneration in the lesion site after SCI (Fan et al., 2018). As our results showed that TUDCA reduced the inflammatory response, thus we considered whether TUDCA could reduce neuron apoptosis and alleviate axon degeneration induced by inflammatory response. After co-cultured with LPS-induced RAW264.7 cell culture supernatant with or without TUDCA for 24 h, TUNEL staining (Figures 3A, B) and immunofluorescence staining of Tuj1 (Figures 3C, D) were performed to evaluate the effect of TUDCA on apoptosis and axon degeneration in spinal neuron primary culture. Sholl analysis was used to quantify the number of branch intersections and distance from the cell body. Our results showed that TUDCA significantly reduced the number of TUNEL positive cells and increased the branch intersection number and length, which suggested that TUDCA treatment could reduce spinal neuron apoptosis and axon degeneration induced by inflammation as compared with control.

### TUDCA reduced tissue damage and promoted wound healing

**FIGURE 4:**
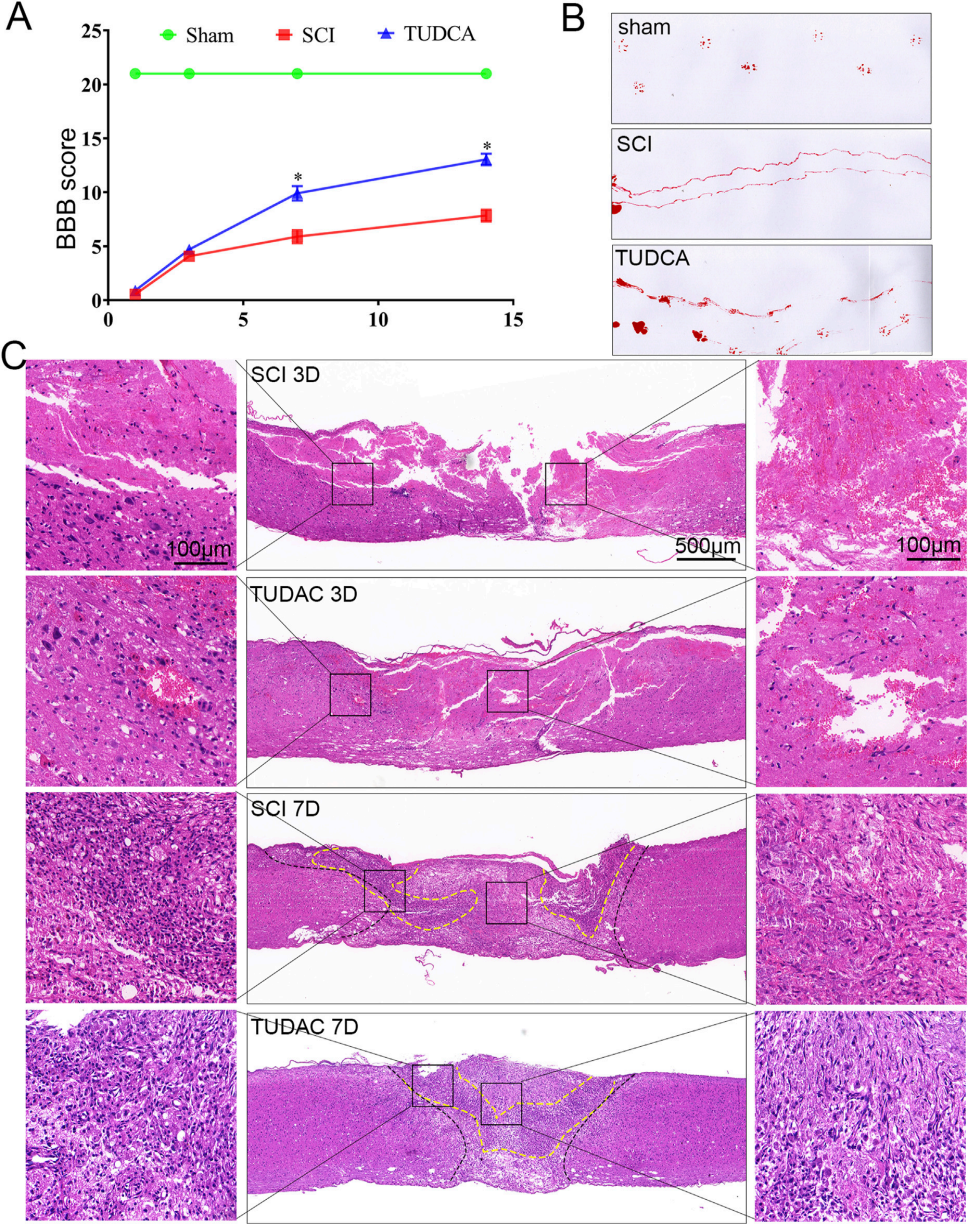
TUDCA treatment improved pathological progress and motor function recovery after SCI. **(A)** The BBB scores of the different groups. **(B)** Footprint analyses of the different groups at day 14 post-injury. **(C)** Reprehensive images from H&E staining in longitudinal Section 3 days and 7 days after injury. All experiments were performed in triplicated and data were presented means ± SEM, n = 3 per group. *P < 0.05 vs. sham group.

The first 7 days after SCI are highly dynamic and are the optimal period for therapeutic intervention (Hu et al., 2023). Therefore, to better exploit the cytoprotective effect of TUDCA in SCI recovery, we first performed Basso-Beattie-Bresnahan (BBB) rating scale (Figure 4A), footprint test (Figure 4B) and H&E staining (Figure 4C) to evaluate the effect of TUDCA on motor function and pathological processes at the early stage of SCI. The BBB scores and the footprint test showed that SCI mice treated with TUDCA showed a relatively locomotor recovery compared with mice in the SCI group. It has been reported that physical trauma to the spinal cord leads to blood invading flow into the cord, neuronal and glial necrosis in the lesion area, axon degeneration along the lesion (Tran et al., 2018). All of these can further lead to cavitation at the lesion site. As shown in Figure 4C, an obvious injured area presented on the spinal cord on day 3 post-injury in SCI and TUDCA groups. Blood cells released by blood vessel rupture, cell debris from necrosis glial, and neurons dominated the lesion center. A few cells were observed at the lesion center in the SCI group; while a few more cells were observed in the TUDCA group, and the amount of blood cells and cell debris decreased. Moreover, at the margin of the injury, cell missing and malformation cavities were observed in the SCI group, while in the TUDCA group tissue was relatively intact. On day 7, blood cells were observed in the lesion epicenter and many more cells migrated to the lesion center in the SCI and TUDCA group. In the rim of the injury, 2 strips of intensive cells (labeled with a dotted yellow line) were observed in the SCI group, while in the TUDCA group, the 2 strips of intensive cells merged into 1 strip and the injured area decreased. This date indicated that TUDCA treatment promoted injury repair by reducing the amount of blood cells in the lesion center and promoting proliferating cell migration to the lesion site.

### The expression changes of genes after TUDCA treatment using RNA sequencing

**FIGURE 5:**
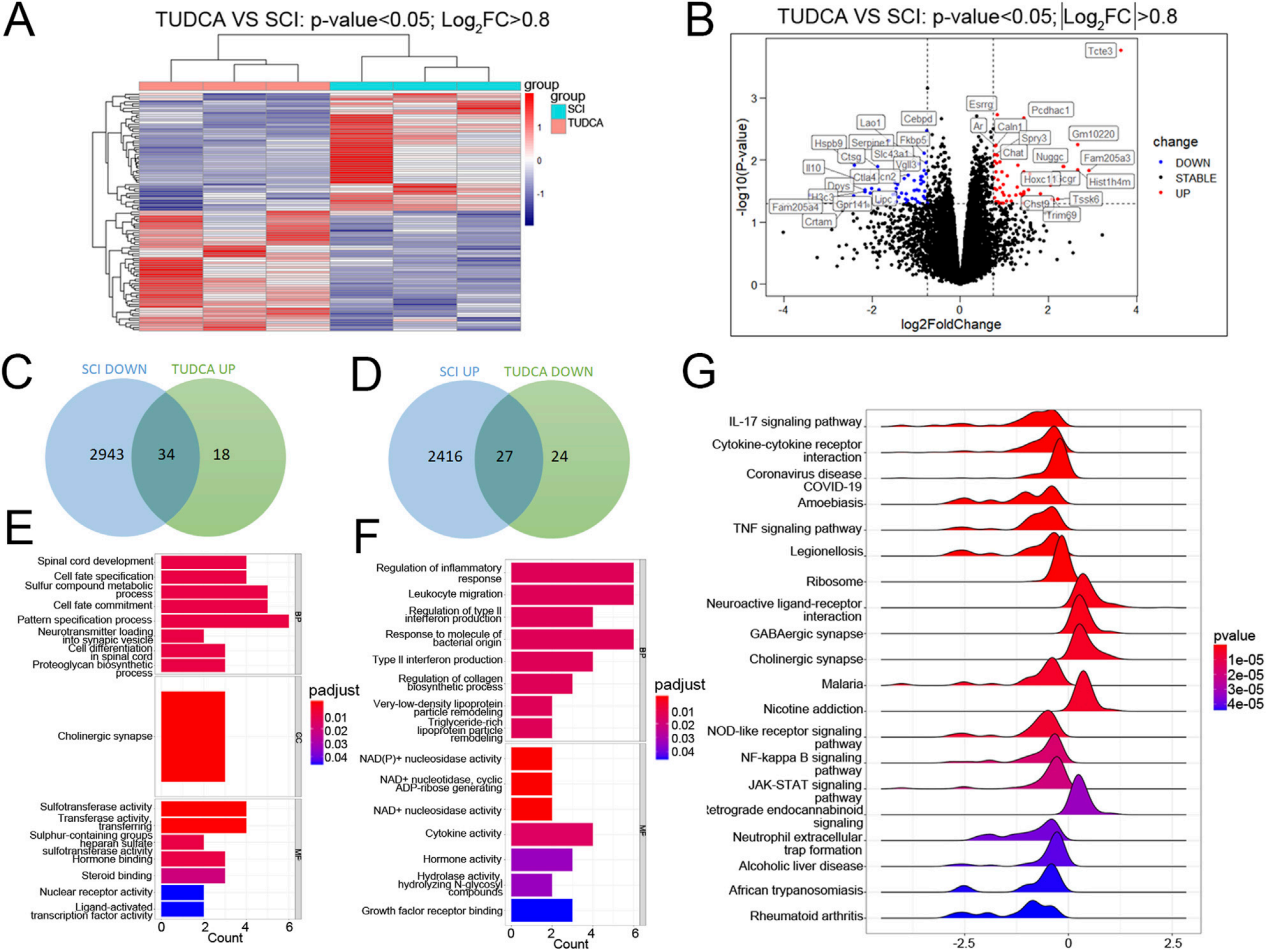
RNA-sequence analysis on the injured spinal cord at day 7 after SCI. **(A)** The heatmap of SCI and TUDCA groups. **(B)** Volcano plot between TUDCA group and SCI controls. **(C, E)** The venn diagram and the enrichment analysis of biological processes about upregulated genes after TUDCA treatment compared to SCI group. **(D, F)** The venn diagram and the enrichment analysis of biological processes about the genes downregulated after TUDCA treatment compared to SCI group. **(G)** Gene Set Enrichment Analysis (GSEA) showed the enriched pathways.

On day 3 after SCI, cell loss presents in the lesion site, and cell debris and blood cells dominate the lesion core. Monocytes infiltrate the spinal cord, differentiate into macrophages in the lesion site, and reach their peak on day 7 (Tran et al., 2018). Meanwhile, reactive astrocytes and endogenous NSCs become activated and migrate toward the lesion core, and their number reaches the peak (Stenudd et al., 2015a). Therefore, the injured spinal cord at day 7 was collected to perform RNA sequencing. As shown in Figure 5A, the heatmap displayed the gene expression changes clustered in SCI and TUDCA groups. Volcano and Venn analysis identified 61 genes (34 genes upregulated and 27 genes downregulated) with significant differences between TUDCA and SCI groups (Figures 5B–D). The Gene Ontology (GO) analysis indicated that the genes upregulated in TUDCA groups involved in biological processes such as spinal cord development and cell differentiation in spinal cord (Figure 5E), and the genes downregulated involved in inflammatory response, leukocyte migration, and type II interferon production (Figure 5F). And the Gene Set Enrichment Analysis (GSEA) showed that cytokine-cytokine receptor interaction, TNF signaling pathway and NF-κB signaling pathway were enriched in TUDCA groups (Figure 5G).

### TUDCA regulated monocyte infiltration and distribution

**FIGURE 6:**
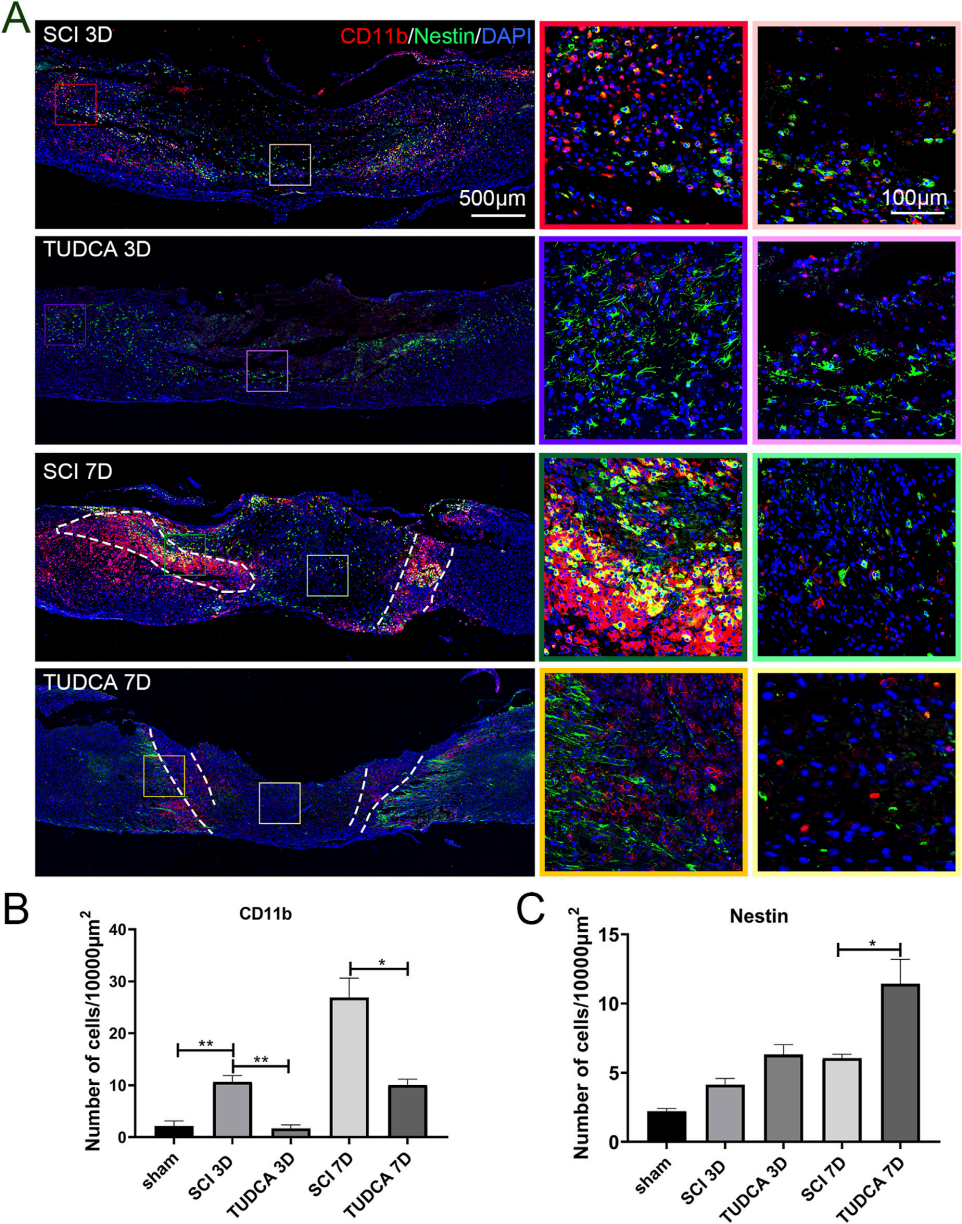
TUDCA regulated monocytes distribution and exerted cytoprotective effects on endogenous NSCs. **(A)** Monocytes visualized using CD11b (red) and endogenous NSCs visualized using Nestin (green) at day 3 and day 7 after SCI. **(B)** Quantification the cell number of CD11b positive monocytes. **(C)** Quantification of the cell number of Nestin positive NSCs. All experiments were performed in triplicated and data were presented means ± SEM, *n* = 3 per group. *P < 0.05, **P < 0.01.

Monocytes in the injured cord are derived from mononuclear phagocytes in the blood. After injuries, monocytes infiltrate the injured cord with the release of cytokines and chemokines by neutrophils, migrate to the site of injury, and differentiate into macrophages (Milich et al., 2019). The first wave starts around 3 days after the injury and peaks around 7 days. As a monocyte marker in mice, CD11b plays an important role in immune regulation, participating in important processes such as inflammatory response and immune cell migration (Wu et al., 2018). Therefore, we performed CD11b and Nestin (NSCs marker) co-immunofluorescence staining to investigate the effect of TUDCA on monocytes and NSCs distribution in SCI mice. On day 3 after the injury (Figure 6A), a few CD11b-positive monocytes and Nestin-positive NSCs migrated to the lesion center of the cord in SCI and TUDCA groups. The cell numbers of CD11b-positive monocytes and Nestin-positive NSCs increased in the rim of the lesion site in the SCI group. However, in the TUDCA group, the cell number of Nestin-positive NSCs also increased in the rim of injury while the cell number of CD11b-positive monocytes decreased compared with the SCI group. On day 7 post-injury, CD11b-positive monocytes migrated to the injury site, formed a strip of intensive monocytes, and separated Nestin-positive NSCs from the uninjured area in the SCI group. While in the TUDCA group, the strip of intensive monocytes was not apparent compared with SCI groups, monocytes showed a sporadic and scattered distribution and interspersed among or in front of Nestin-positive NSCs. Statistical analyses were performed on the cell numbers of CD11b-positive monocytes and Nestin-positive NSCs in the rim of the injury between SCI and TUDCA groups (Figures 6B, C). After the injury, the cell number of CD11b-positive monocytes increased significantly on day 3 and day 7 in SCI groups and decreased significantly in the TUDCA group. Meanwhile, the cell number of Nestin-positive NSCs significantly increased only on day 7 post-injury in the TUDCA group.

**FIGURE 7:**
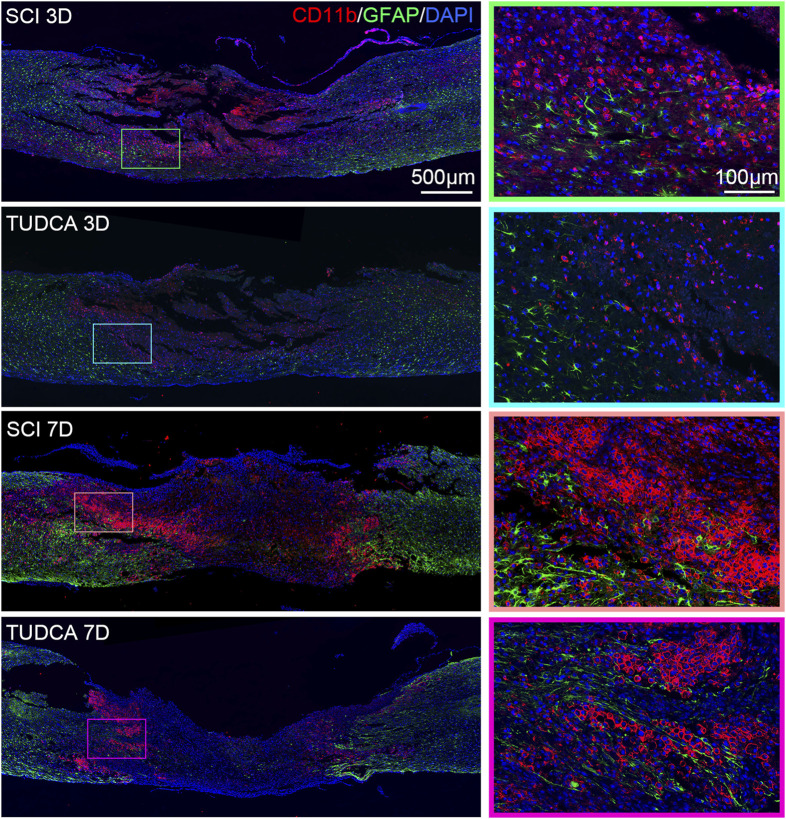
TUDCA regulated monocytes distribution and impacted glial scar formation. Co-immunofluorescence images showed reactive astrocytes (GFAP, green) and monocytes (CD11b, red) at day 3 and day 7 after SCI.

Within the influx of myeloid cells, astrocytes become activated at the lesion penumbra and begin to migrate to the lesion margin. The astrocytes reactive proliferation and migration help to surround the lesion area, resolve the inflammation, and form the glial scar (Orr and Gensel, 2018). Therefore, we performed CD11b and GFAP (astrocytes marker) co-immunofluorescence staining to investigate the effect of TUDCA on monocyte distribution and glial scar formation at the early stage of SCI. As shown in Figure 7, GFAP-positive astrocytes encircled the monocytes in the lesion area at day 3 after injury in SCI and TUDCA groups. On day 7 post-injury, reactive astrocyte migration was retained by the strip of intensive monocytes in the SCI group. In the TUDCA group, because the cell number of monocytes decreased, astrocytes penetrated the monocyte block and migrated to the lesion area.

In summary, these results indicated that TUDCA treatment regulated monocyte infiltration and distribution to affect endogenous NSCs and reactive astrocyte migration at the early stage after SCI.

### TUDCA regulated macrophage distribution

**FIGURE 8:**
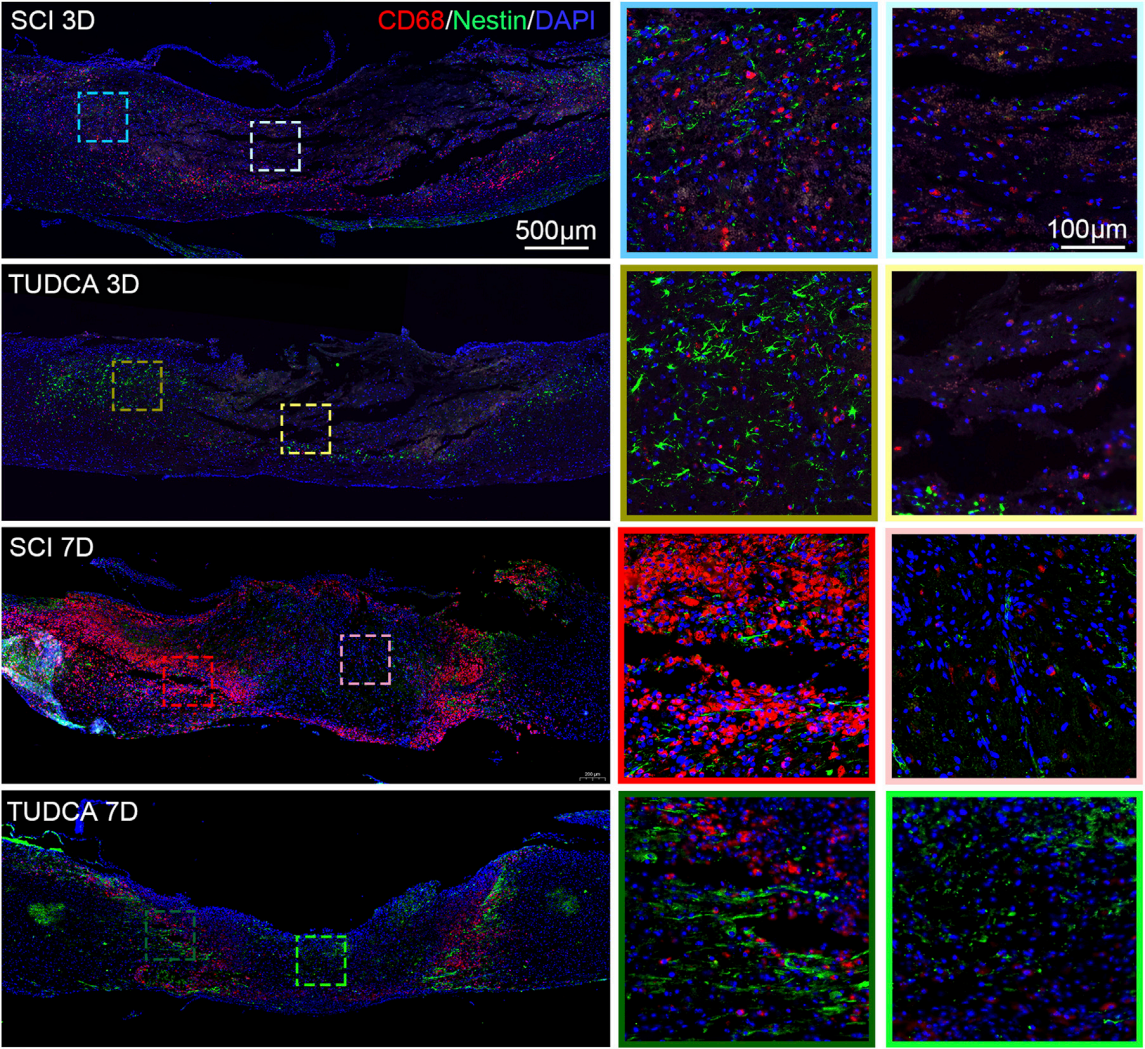
TUDCA regulated macrophages and endogenous NSCs distribution. Co-immunofluorescence images showed macrophages (CD68, red) and endogenous NSCs (Nestin, green) at day 3 and day 7 after SCI.

**FIGURE 9:**
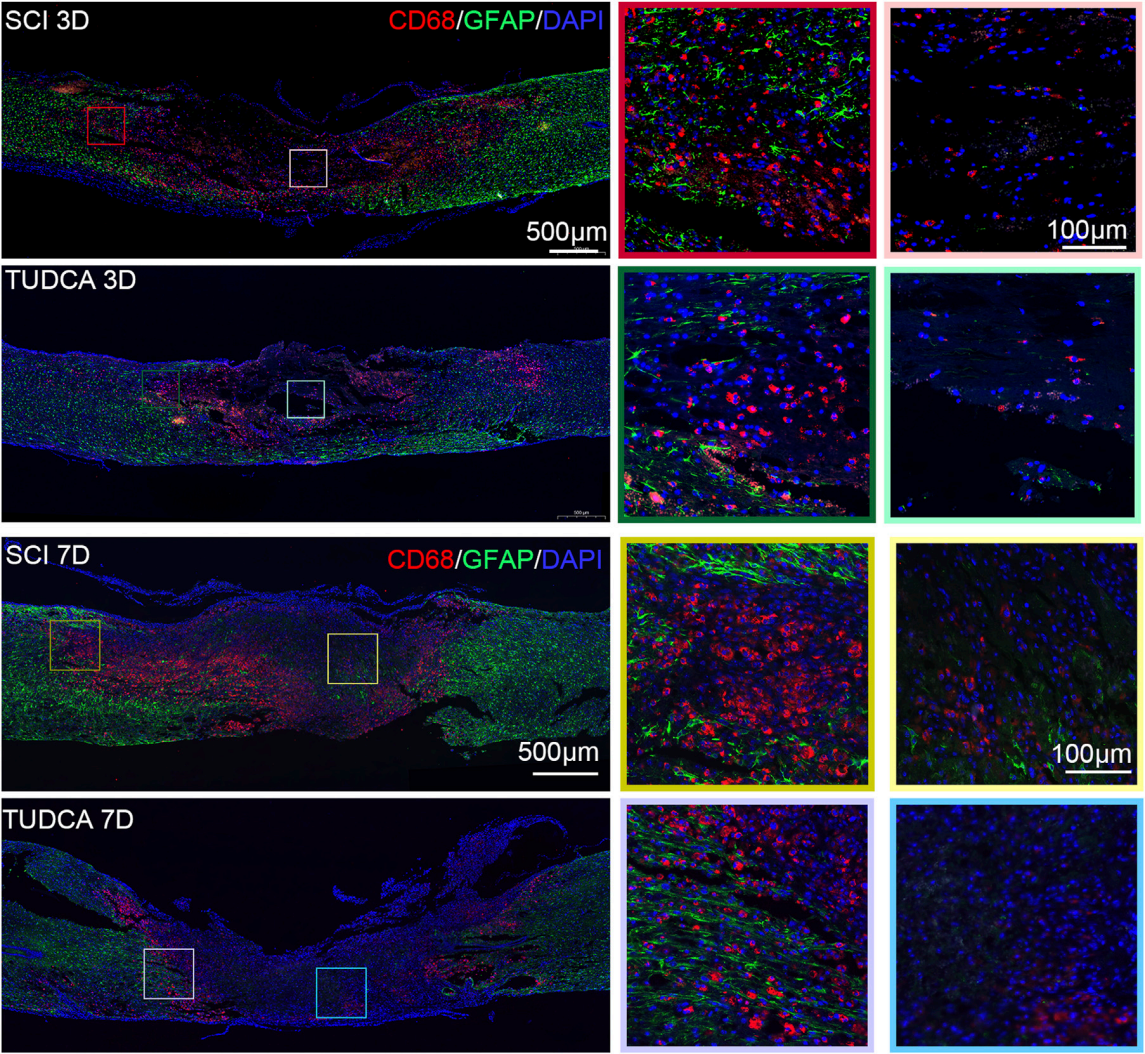
TUDCA regulated macrophages and reactive astrocytes distribution. Co-immunofluorescence images showed macrophages (CD68, red) and reactive astrocytes (GFAP, green) at day 3 and day 7 after SCI.

After infiltrating the cord, blood-borne monocytes differentiate into macrophages in the injury cord and the number reaches the first peak at day 7 post-injury. Macrophages in the lesion site not only phagocytize cell debris and blood cells but also release pro-inflammatory factors which may lead to neuronal and glial cell death and tissue damage expansion (Van Broeckhoven et al., 2021). To investigate the effect of TUDCA on macrophage distribution, NSCs migration, and glial scar formation, we performed CD68 (an active macrophage marker) and Nestin (Figure 8), CD68 and GFAP (Figure 9) immunofluorescence staining in SCI mice. Macrophages showed a similar distribution as monocytes on day 3 and day 7 in the SCI and TUDCA groups. In SCI groups, CD68-positive macrophages were observed at the rim of the lesion site, interweaved with NSCs on day 3 post-injury, and aggregated to form an intensive cell strip at the two sides of the lesion site on day 7. While in TUDCA groups, the cell numbers of macrophages decreased on day 3 and day 7, and many more NSCs were observed at the margin of the lesion site at day 3 and day 7 post-injury (Figure 8). GFAP-positive reactive astrocytes encircle the lesion site on day 3 in SCI and TUDCA groups. On day 7, an intensive cell strip at the margin of the lesion site prevented reactive astrocyte migration which restricted the glial formation. In the TUDCA group, reactive astrocytes migration was not retained by scattered macrophages on day 7 (Figure 9). All these results showed that TUDCA treatment regulated monocytes/macrophage distribution to provide a beneficial microenvironment for endogenous NSCs and reactive astrocytes migration.

### TUDCA transferred macrophages from M1 to M2 phenotypes and altered endogenous NSCs morphology

**FIGURE 10:**
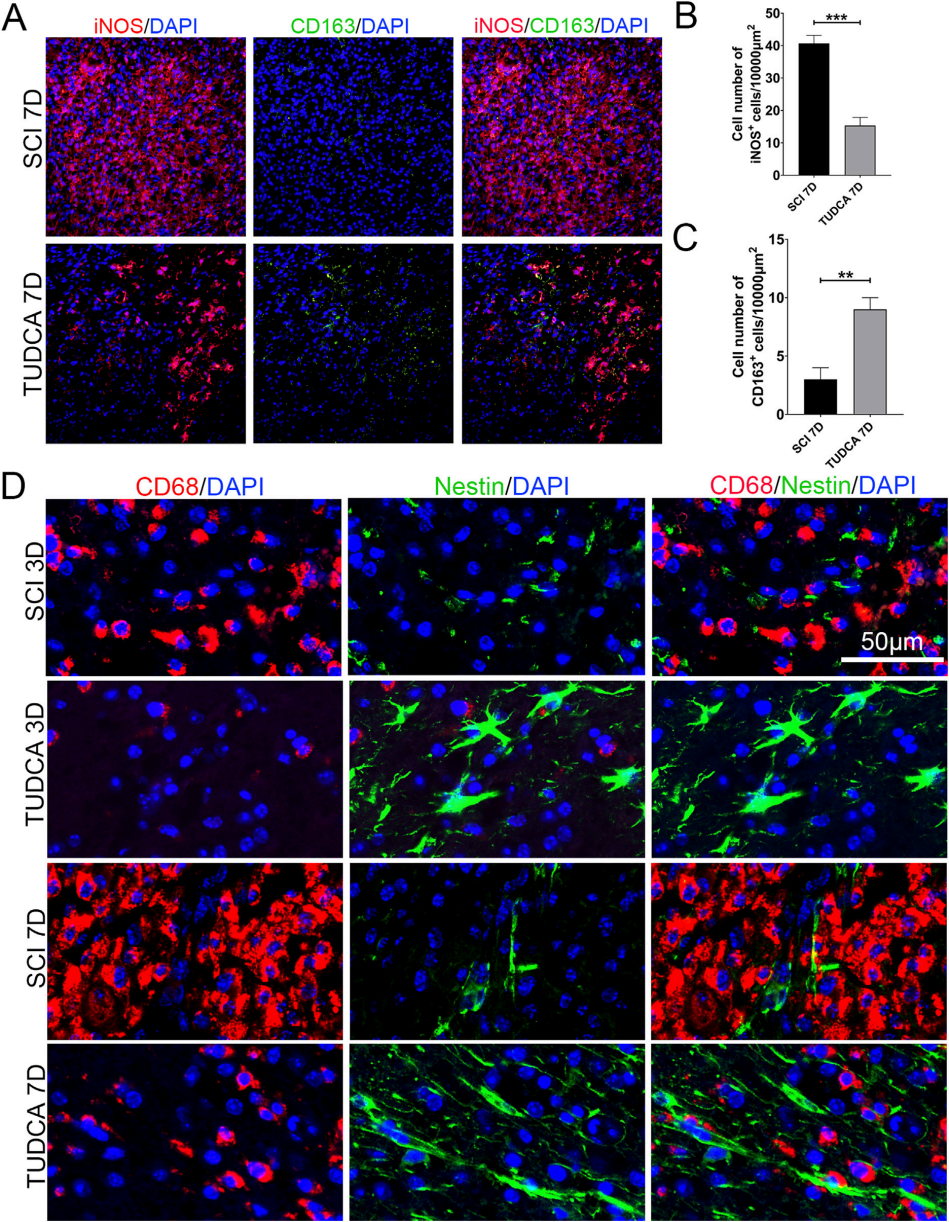
TUDCA promoted macrophages shift to M2-like phenotype and impacted endogenous NSCs morphology. **(A)** Immunofluorescent staining of M1-like (iNOS, red) and M2-like macrophages (CD163, green) at the margin of the lesion site at day 7 after SCI. **(B, C)** Quantification the number of iNOS^+^ or CD163 cells. **(D)** Macrophages (CD68, red) and endogenous NSCs (Nestin, green) at the margin of the lesion site at day 7 after SCI. All experiments were performed in triplicated and data were presented means ± SEM, *n* = 3 per group. **P < 0.01, ***P < 0.001.

Previous studies have reported that different macrophage phenotypes play different roles in inflammatory response, classically M1 macrophages release proinflammatory cytokines and M2 macrophages release anti-inflammatory cytokines and hasten wound healing (Van Broeckhoven et al., 2021). We performed iNOS (M1-associated marker) and CD163 (M2-associated marker) immunofluorescent staining to assess the phenotype of macrophages. INOS-positive macrophages at the margin of the lesion were significantly increased and most of them were CD163 negative at day 7 after SCI (Figures 10A–C). Compared with the SCI group, the cell number of macrophages significantly decreased and the cell number of CD163 positive significantly increased after TUDCA treatment. It has been reported that microenvironment imbalance of the cord impacts endogenous NSC migration and differentiation (Fan et al., 2018). Therefore, we observed the cell morphology alteration of NSCs on day 3 and day 7 with TUDCA treatment after SCI Figures 10A–D. A few endogenous NSCs labeled with Nestin presented among macrophages and were restricted by macrophages in SCI group. In TUDCA groups, NSCs appeared as astrocyte morphology on day 3 and showed an obvious migration tropism on day 7. These indicated that TUDCA treatment provided a beneficial microenvironment for endogenous NSCs by reducing macrophage cell number and promoting macrophage conversion into M2-like phenotypes.

### TUDCA treatment promoted neuron regeneration after SCI

**FIGURE 11:**
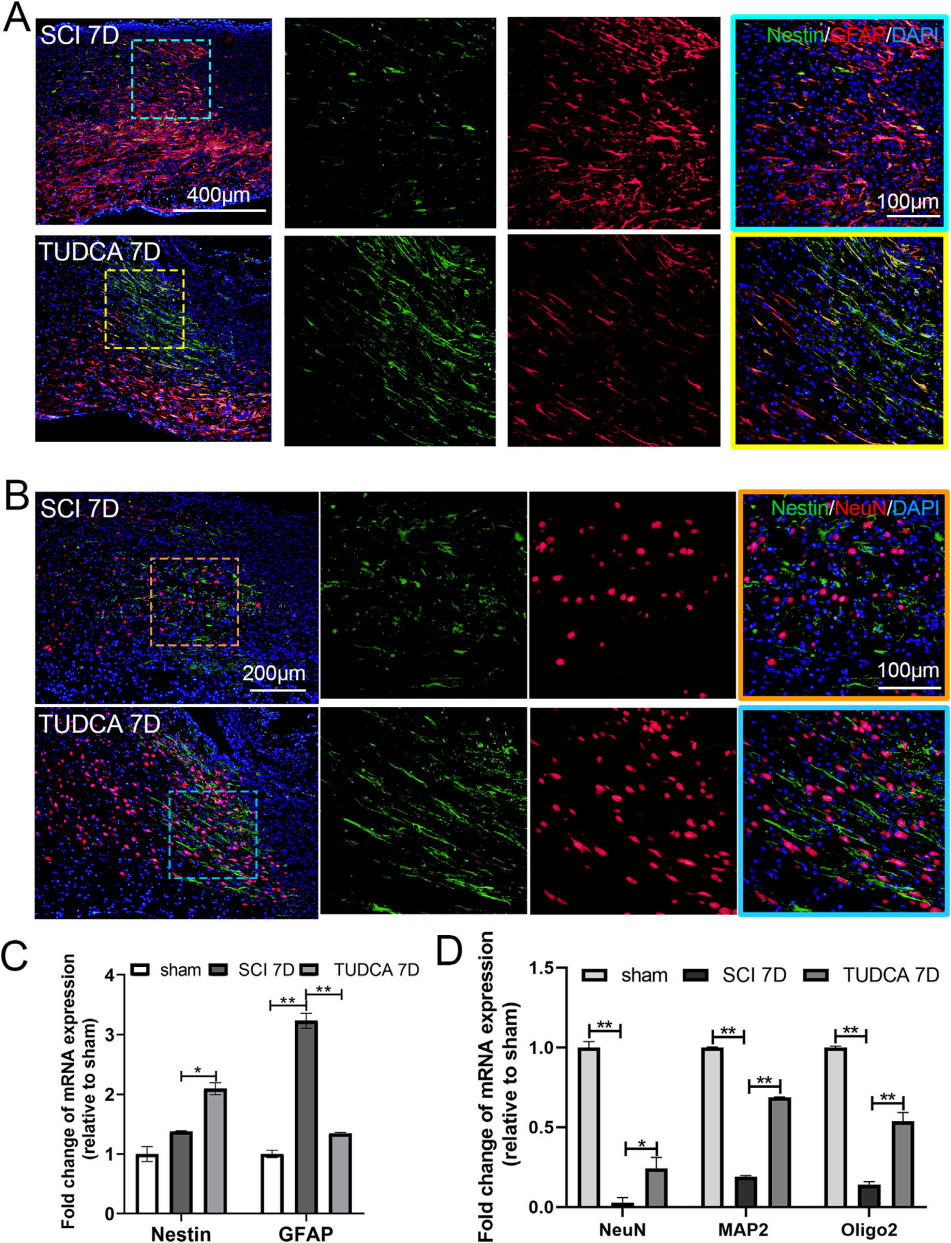
TUDCA promoted neuron regeneration along endogenous NSCs migration at day 7 after SCI. **(A)** Co-immunofluorescence showed endogenous NSCs (Nestin, green) and reactive astrocytes (GFAP, red) at the margin of the lesion site at day 7 after SCI. **(B)** Endogenous NSCs (Nestin, green) and neuron (NeuN, red) at the margin of the lesion site at day 7 after SCI. **(C, D)** Quantitative polymerase chain reaction (qPCR) showing the expression of Nestin, GFAP, NeuN, MAP2 and Oligo 2 at day 7 after SCI. All experiments were performed in triplicated and data were presented means ± SEM, *n* = 3 per group. *P < 0.05, **P < 0.01.

Astrocytes reactive action, endogenous NSCs proliferation and migration, and neuron regeneration in the injured spinal cord are crucial for motor function recovery after SCI (Anjum et al., 2020). So we performed immunofluorescent staining and quantitative polymerase chain reaction (qPCR) to elaborate the astrocytes’ reactive action, endogenous NSCs proliferation, and neuron regeneration in SCI mice with TUDCA treatment. Compared with the sham group, GFAP-positive astrocytes were increased and a few Nestin-positive NSCs were observed near the lesion site on day 7 in the SCI group (Figure 11A). Whereas after TUDCA treatment, GFAP-positive astrocytes reduced and Nestin-positive NSCs increased. qPCR results also showed that TUDCA treatment inhibited GFAP mRNA expression and increased Nestin mRNA expression (Figure 11C). It has been reported endogenous NSCs migrate to the lesion site after SCI and differentiate into astrocytes, oligodendrocytes, and neurons (Stenudd et al., 2015b). Neuronal Nuclei (NeuN) has been widely used as a marker of post-mitotic neurons in the central nervous system (Duan et al., 2016). Immunostaining of NeuN and Nestin was used to illustrate the NSCs distribution and nerve regeneration at the margin of the lesion site on the day 7 after SCI. After TUDCA treatment, NeuN-positive cells were observed and cell number increased at the margin of the lesion site on day 7 (Figure 11B). The qPCR results also showed that NeuN, microtubule-associated protein 2 (MAP2, a neuron-specific cytoskeletal protein), and Oligo2 (a marker of oligodendrocyte) mRNA expression were restored at day 7 by TUDCA treatment after SCI (Figure 11D). All these data indicated that TUDCA treatment could promote neuron regeneration in the lesion site.

## Discussion

TUDCA, as a neuroprotective hydrophilic bile acid, has been reported to show a neuroprotective effect by regulating apoptosis-related proteins such as Bax, Bcl2, and caspase3 and reducing inflammatory response in different SCI animal models (Kim et al., 2018; Zhang et al., 2018). However, the underlying mechanism of TUDCA on regulating monocytes/macrophages to impact inflammatory response after SCI has not been elucidated. Following SCI, resident microglia in the cord are activated and engaged in inflammation in response to injury immediately. Meanwhile, monocytes infiltrate the cord, differentiate into macrophages, and dominate the lesion site within 3–7 days, while microglia are rapidly lost in the lesion core (Zrzavy et al., 2021). According to the pivotal role of monocytes/macrophages in inflammatory response, we considered that TUDCA may regulate monocyte filtration and macrophage distribution to impact inflammatory response after SCI. The inflammatory microenvironment in the cord after SCI is a determinant of neuron survival and endogenous NSC fate (Hellenbrand et al., 2021). In this study, we first demonstrated that TUDCA treatment regulated monocyte/macrophage distribution and impacted endogenous NSCs morphological changes and migration to promote wound healing and rehabilitation in SCI mice.

In order to exploit the neuroprotective role of TUDCA treatment in SCI mice, first we hypothesized that TUDCA regulated inflammation to impact spinal NSCs migration and proliferation, spinal neuron survival, and axon degeneration *in vitro*. Our results showed that TUDCA treatment reduced inflammation by inhibiting NF-κB signaling pathway, and then restored spinal NSC migration and proliferation restrained, and increased their survival *in vitro*. TUDCA also increased spinal neuron survival and alleviated axon degeneration caused by inflammation. All of these were consistent with our hypothesis, and then we detected the neuroprotective role of TUDCA treatment in SCI mice.

The first 7 days are highly dynamic and complicated, including blood cell infiltration, neuron and glial cell death, immune cell infiltration, endogenous NSCs activation and migration, astrocyte reactivation, and so on (Zrzavy et al., 2021). It is also the optimal time for pharmacological and surgical intervention for SCI. Therefore, we aim to illustrate the effect and related mechanism of TUDCA on inflammatory response at the subacute stage after SCI. We checked the effect of TUDCA on the pathological progress after SCI using H&E staining. It was shown that TUDCA treatment improved the pathological progress and promoted wound healing. Besides, RNA sequencing analysis showed that genes downregulated by TUDCA treatment were involved in the regulation of inflammatory response, leukocyte migration, and type II interferon production, while genes upregulated were involved in spinal cord development and cell differentiation in the spinal cord. These results illustrated that TUDCA promoted wound healing by regulating the inflammatory response.

Finally, we further investigated monocyte/macrophage distribution and its impact on endogenous NSCs and reactive astrocyte migration in SCI mice. On day 7 after injury, monocytes/macrophages dominated the lesion site and formed an intensive cell barrier that separated endogenous NSCs from reactive astrocytes and restricted the migration of endogenous NSCs and reactive astrocytes toward the lesion core. Endogenous NSCs and reactive astrocytes increase cell proliferation, migrate towards the lesion, and contribute to glia scar formation which can limit damage expansion and accelerate wound healing (Orr and Gensel, 2018; Perez-Gianmarco and Kukley, 2023).

Besides, endogenous NSCs showed multi-lineage differentiation, mostly giving rise to astrocytes and a few neurons (Gilbert et al., 2022). Inflammatory microenvironment in the spinal cord is the determining factor for regeneration and recovery after SCI (Freyermuth-Trujillo et al., 2022). Therefore, regulation of inflammatory response to provide a favorable microenvironment for reactive astrocyte migration, endogenous NSCs migration, and differentiation is an effective therapeutic strategy for SCI therapy. TUDCA treatment significantly decreased the number of monocytes/macrophages and altered their distribution. Meanwhile, macrophages underwent a shift from classic M1 to M2-like phenotypes after TUDCA treatment, suggesting that TUDCA exert its therapeutic effect by reversing the polarization status of macrophage (Wang et al., 2023). As the inflammatory microenvironment changed, TUDCA treatment increased the cell number of endogenous NSCs with normal morphology and migration tropism to the lesion site. Glial scar helps to resolve inflammation to some extent, but a permanent glial scar generates an intensive barrier for nerve regeneration (Clifford et al., 2023). TUDCA treatment showed a moderate regulation of astrocyte reactivation and prevented their overactivation to restrict the glial scar area. Besides, the immunofluorescent staining of Nestin and NeuN showed that TUDCA treatment promoted nerve regeneration to reduce nerve loss caused by inflammation. Therefore, TUDCA showed neuroprotective roles in inflammatory response regulation and glial scar formation after SCI.

All of these demonstrated that TUDCA treatment decreased monocytes/macrophages and altered their distribution to provide a conductive microenvironment for tissue repair and nerve regeneration in the lesion site. However, the specific mechanism of TUDCA on monocytes/macrophages and combined therapies might be explored in the future.

## Conclusion

In conclusion, this study demonstrated that TUDCA restored spinal NSC migration and proliferation and reduced spinal NSCs and neuron apoptosis and axon degeneration by regulating inflammatory response *in vitro*. TUDCA treatment regulated monocyte/macrophage distribution and improved the microenvironment to promote nerve regeneration in SCI mice. TUDCA improved the microenvironment of the spinal cord by significantly decreasing the number of monocytes/macrophages, altering their distribution, and promoting macrophages shift to M2-like phenotypes. Our findings provide evidence that TUDCA treatment is an effective intervention at the subacute stage of SCI, and the effect of TUDCA in the chromic stages needs to be explored in future studies.

## Data Availability

The datasets presented in this study can be found in online repositories. The names of the repository/repositories and accession number(s) can be found below: https://ngdc.cncb.ac.cn/, CRA021821.

## Abbreviations

BrdU, Bromodeoxyuridine; DMEM, Dulbecco’s modified Eagle medium; GFAP, Glial fibrillary acidic protein; FBS, Fetal bovine serum; LPS, Lipopolysaccharide; MAP2, Microtubule Associated Protein 2; NeuN, Neuronal nuclei; NSCs, Neural stem cells; PFA, Paraformaldehyde; SCI, Spinal cord injury; TUDCA, Tauroursodeoxycholic acid; Tuj1, neuron-specific class III beta-tubulin.
