# Supplementary material for: Tauroursodeoxycholic acid regulates macrophage/monocyte distribution and improves spinal microenvironment to promote nerve regeneration through inhibiting NF-κB signaling pathway in spinal cord injury
Source: Front Pharmacol. 2025 Apr 10;16:1554945. doi: 10.3389/fphar.2025.1554945 (PMC12019990; doi:10.3389/fphar.2025.1554945)
Supplement: Supplementary file 1 [file DataSheet1.docx]

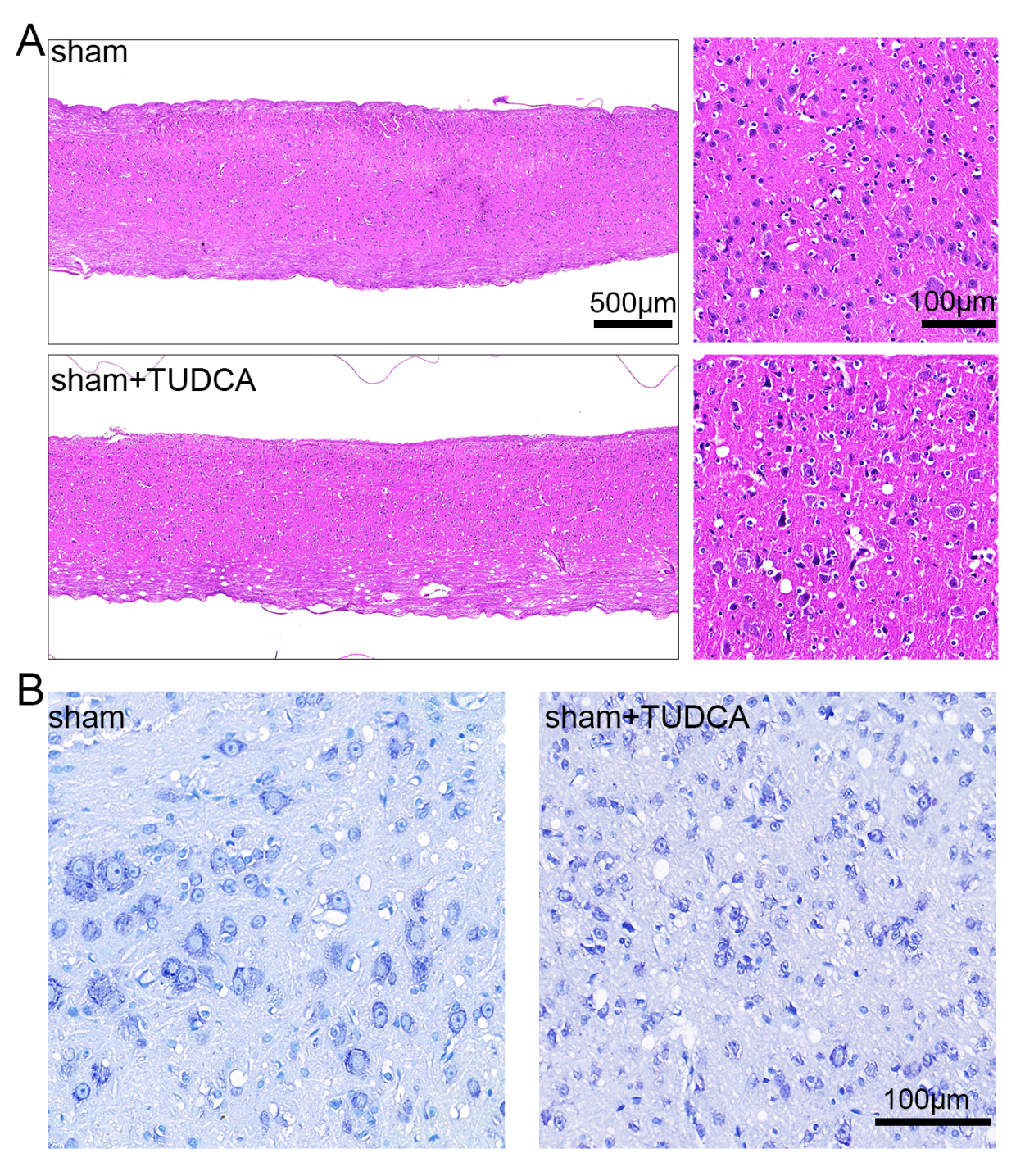


**Figure S1. The histological morphology and the normal neurons in normal mice treated with TUDCA.** (A) Representative images from H&E staining in longitudinal section. (B) The survived neurons were stained by Nissl Staining.


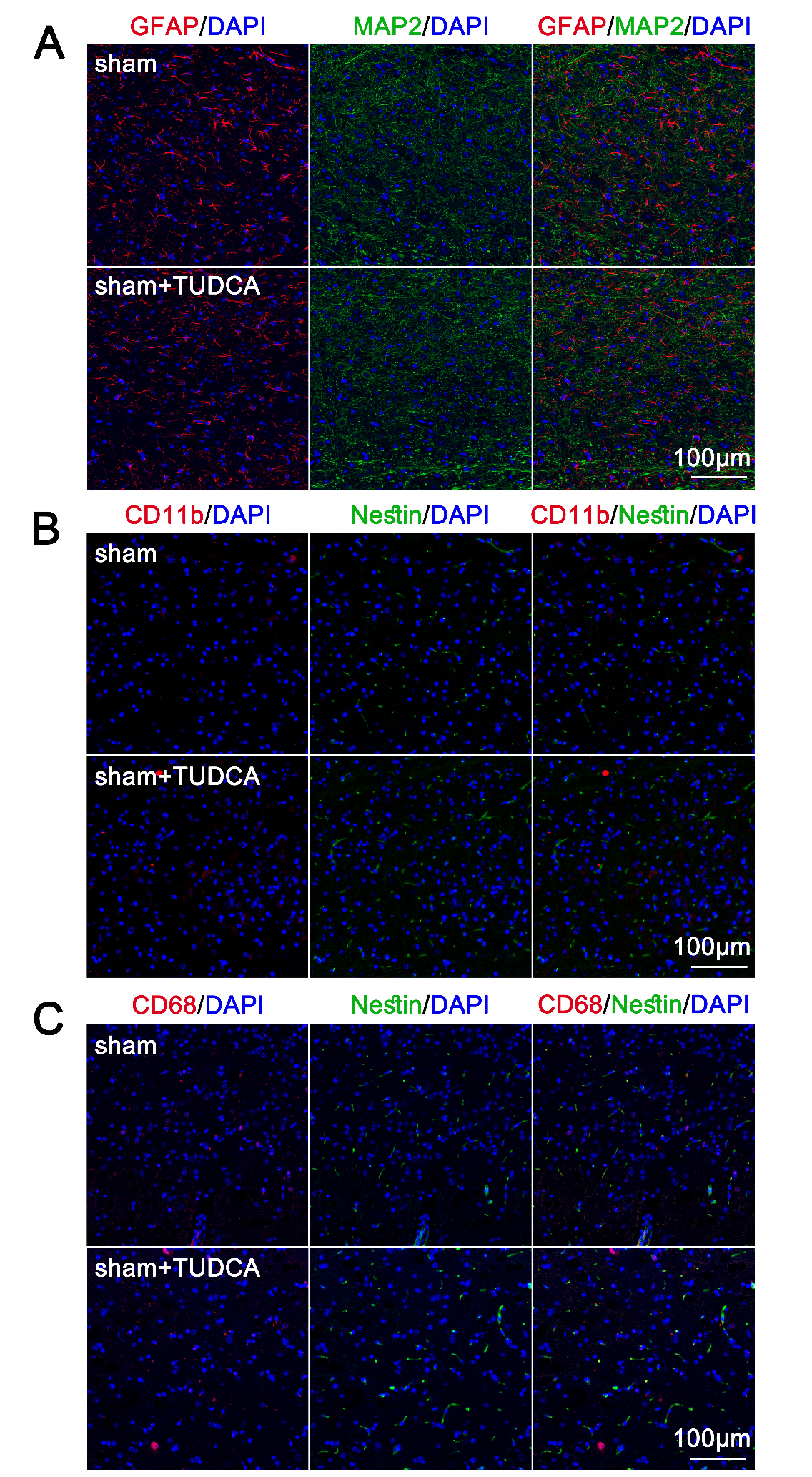


**Figure S2. The distribution of astrocytes and neurons, macrophages and NSCs in normal mice treated with TUDCA.** **(A)** Co-immunofluorescence images showed the distribution of astrocytes (GFAP positive, red) and neurons (MAP2 positive, green) in normal mice treated with TUDCA for 14 days. **(B，C)** Co-immunofluorescence images showed the distribution of macrophages (CD11b positive or CD68 positive, red) and NSCs (Nestin positive, green) in normal mice treated with TUDCA for 14 days.
